# Distinct patterns of gene and protein expression elicited by organophosphorus pesticides in *Caenorhabditis elegans*

**DOI:** 10.1186/1471-2164-10-202

**Published:** 2009-04-29

**Authors:** John A Lewis, Maria Szilagyi, Elizabeth Gehman, William E Dennis, David A Jackson

**Affiliations:** 1 US Army Center for Environmental Health Research, Fort Detrick, MD, USA; 2 US Environmental Protection Agency, Washington, DC, USA; 3 Battelle National Biodefense Institute, Frederick, MD, USA

## Abstract

**Background:**

The wide use of organophosphorus (OP) pesticides makes them an important public health concern. Persistent effects of exposure and the mechanism of neuronal degeneration are continuing issues in OP toxicology. To elucidate early steps in the mechanisms of OP toxicity, we studied alterations in global gene and protein expression in *Caenorhabditis elegans *exposed to OPs using microarrays and mass spectrometry. We tested two structurally distinct OPs (dichlorvos and fenamiphos) and employed a mechanistically different third neurotoxicant, mefloquine, as an out-group for analysis. Treatment levels used concentrations of chemical sufficient to prevent the development of 10%, 50% or 90% of mid-vulval L4 larvae into early gravid adults (EGA) at 24 h after exposure in a defined, bacteria-free medium.

**Results:**

After 8 h of exposure, the expression of 87 genes responded specifically to OP treatment. The abundance of 34 proteins also changed in OP-exposed worms. Many of the genes and proteins affected by the OPs are expressed in neuronal and muscle tissues and are involved in lipid metabolism, cell adhesion, apoptosis/cell death, and detoxification. Twenty-two genes were differentially affected by the two OPs; a large proportion of these genes encode cytochrome P450s, UDP-glucuronosyl/UDP-glucosyltransferases, or P-glycoproteins. The abundance of transcripts and the proteins they encode were well correlated.

**Conclusion:**

Exposure to OPs elicits a pattern of changes in gene expression in exposed worms distinct from that of the unrelated neurotoxicant, mefloquine. The functional roles and the tissue location of the genes and proteins whose expression is modulated in response to exposure is consistent with the known effects of OPs, including damage to muscle due to persistent hypercontraction, neuronal cell death, and phase I and phase II detoxification. Further, the two different OPs evoked distinguishable changes in gene expression; about half the differences are in genes involved in detoxification, likely reflecting differences in the chemical structure of the two OPs. Changes in the expression of a number of sequences of unknown function were also discovered, and these molecules could provide insight into novel mechanisms of OP toxicity or adaptation in future studies.

## Background

The wide use of organophosphorus (OP) based pesticides and unresolved issues in their toxicity, including the causes of persistent and off-target effects and the mechanisms of neuronal degeneration, make them an important concern for public health. OPs are a class of chemicals that inhibit serine esterases by covalently bonding with the active site serine. Two primary targets of OPs have been implicated in human toxicity, acetylcholinesterase (AChE; reviewed in [1]) and neuropathy target esterase (NTE; reviewed in [2]). However, the inhibition of AChE is of more concern because of acetylcholine's role as a neural transmitter. Long-term adverse effects of OP exposure have been described [3-5], but the nature and mechanism of persistent effects are relatively poorly understood.

The principal risk of toxicity from OPs and other AChE inhibitors occurs after high level, acute exposures when death from respiratory failure may rapidly ensue; less severe exposures may cause salivation, lacrimation, incontinence, and convulsions followed by paralysis potentially resulting in death (reviewed in [6,7]). However, a number of persistent and delayed effects of OP exposure are also known. A so-called intermediate syndrome–defined by weakness of the neck, proximal limb, and respiratory musculature–may present 24–96 hours after exposure and is believed to be the result of acetylcholine receptor desensitization (reviewed in [1,8]). Organophosphate induced delayed polyneuropathy (OPIDP) is a delayed syndrome (7–21 days after exposure) that is characterized by numbness, weakness, and paresthesia in the limbs and degeneration of peripheral nerves and central nervous system myelin sheaths; inhibition of NTE is thought to underlie OPIDP (reviewed in [1,8,9]). Chronic neurological and neuropsychiatric effects–some of which may persist for years–and developmental neuro-behavioral effects have also been described [10-12].

In an effort to understand the mechanisms of OP toxicity, we have tracked global gene and protein expression after intoxication by two OPs, dichlorvos and fenamiphos, using the genomic model organism *Caenorhabditis elegans *with whole genome microarrays and mass spectrometry-based proteomics. We selected two chemically different OPs to ask whether it is possible to distinguish between the biological responses to different inhibitors of AChE. To discriminate generalized alterations in gene expression due to neurotoxicity and stress from OP specific effects, we included a third chemical, mefloquine, as an out-group. Mefloquine is believed to cause neurotoxicity by perturbing Ca^++ ^homeostasis, most likely through interference with an ion channel [13,14].

Using *C. elegans *for toxicological studies provides a number of benefits. The organism is well studied, has a very simple body plan, and has a completely sequenced genome. Further, the responses of *C. elegans *to a number of toxicants have been shown to resemble those of mammals in a number of cases ranging from anesthetics to metals to OP pesticides [15-21] (see also [22] for a recent review of the uses of *C. elegans *in toxicological research), and the availability of commercial microarrays has facilitated the investigation of the mechanism of action of an array of toxicants at the functional genomic level (*e.g*., [23-25]).

*C. elegans *does not require neuronal signals for respiration and is very resistant to death via OP intoxication yet shows substantial similarity to mammals in the relevant biochemistry and genomics [20]. The acute toxicity of OP pesticides results from inhibition of AChE in vertebrates [26] and in nematodes [20]. The *C. elegans *genome also contains two homologs of the vertebrate secondary OP target, NTE (ZK370.4 and M110.7; [27] and unpublished observations). While it is unknown whether inhibition of either of the *C. elegans *NTE homologs will induce an OPIDP-like condition, the syndrome has been described in humans following dichlorvos exposure (reviewed in [28]) raising the possibility that dichlorvos might be a suitable compound for investigating this effect. Furthermore, because *C. elegans *is resistant to OP lethality, we reasoned that by using this organism to study the effects of dichlorvos and fenamiphos, it might be possible to expose the worms to high doses of OPs to highlight changes in gene and protein expression that are difficult to discern using classical methods or animal models that are less resistant to OPs.

A drawback to using *C. elegans*, however, is that the worms are usually cultured with bacteria as food source [29]. The presence of bacteria may complicate the interpretation of data because of the metabolism of test materials by the feeder organisms and the contamination of protein and nucleic acid samples with bacterial molecules. While a number of axenic media have been previously described (for example [30-36]), nematodes cultured in axenic media have generally shown reduced rates of development and extended life-spans, suggesting that the media lack essential nutrients. To overcome this problem, we developed a defined, liquid, sterile medium (CeHR medium) [37] in which *C. elegans *can be stably propagated with a generation time similar to that of worms on bacterial plates [37,38].

In this study, we exposed developmentally synchronized *C. elegans *cultures in CeHR medium to two structurally different OPs, dichlorvos and fenamiphos, and the functionally dissimilar neurotoxicant, mefloquine, as an out-group. Global gene expression was determined by microarray analysis of RNA from harvested worms, and proteins extracted from parallel worm cultures were analyzed by mass spectrometry to identify changes in protein expression. Proteomic and functional genomic analysis revealed sets of genes and proteins that distinguish not only between exposure to the OPs and to mefloquine, but also between the OPs themselves. The results are generally consistent across the transcriptomic and proteomic analyses and can readily be understood in the context of the known effects of OP intoxication.

## Methods

### Nematode culture

*C. elegans *[N2 wild type, DR subclone of CB original (Tcl pattern I), obtained from Caenorhabditis Genetics Center] were maintained in synchronized cultures grown in CeHR medium (see below). All cultures were grown at 22.5°C with shaking at 70 rpm on an Innova 2000 platform shaker (New Brunswick Scientific, Edison, NJ). Typically, 5 × 10^5 ^L1 larvae were used to inoculate 40 mL of medium in a T-75 flask. Stock cultures were propagated using the synchronization procedure described below to ensure that sufficient numbers of developmentally synchronized worms were available for experimentation at all times. CeHR medium is a sterile, defined medium, supplemented with 20% (v/v) ultrapasteurized organic, fat-free milk for the axenic propagation of *C. elegans*. A detailed description of the preparation of the medium is available from the USACEHR on request and in [37].

### Synchronization of cultures

Embryos were isolated using a minor modification of the bleaching method of Stiernagle [39] described by Szilagyi *et al*. [37]. The isolated embryos were suspended in 30 mL M9 buffer (42.3 mM Na_2_HPO_4_, 22.0 mM KH_2_PO_4_, 85.6 mM NaCl, 1 mM MgSO_4_), transferred into T-75 culture flasks and incubated at 22.5°C overnight to allow hatching and arrest at the L1 stage. L1 larvae were used within three days to start developmentally synchronized cultures.

### Rangefinding

A developmental inhibition assay was used to determine exposure concentrations. Synchronized worms grown at 22.5°C with shaking at 70 rpm progress from the mid-vulval L4 larval stage to the early gravid adult (EGA) stage within 24 h. The presence of toxicants inhibits this development. To determine concentrations corresponding to effect concentrations (EC) of EC_10_, EC_50_, and EC_90_, (concentrations preventing 10%, 50%, and 90% of the worms from developing to EGA), 8 × 10^4 ^L1 larvae were inoculated into T-25 flasks–each containing 10 mL of CeHR medium. When 90% of the worms had developed to mid-vulval L4 larvae (44–46 h), chemical was added. The flasks were incubated for 24 h, after which a sample of worms was examined microscopically to assess their developmental stage. The toxicant concentrations corresponding to EC_10_, EC_50_, and EC_90 _were selected for the exposure experiments (Table 1).

**Table 1:** Concentrations of test chemicals

Chemical	Developmental Arrest (%)	Nominal Conc. (mg/L)	Average Conc. (mg/L)	Rep 1 Conc. (mg/L)	Rep 2 Conc. (mg/L)	Rep 3 Conc. (mg/L)
	10	3	3.55	3.65	3.44	3.57
dichlorvos	50	15	16.0	16.5	15.9	15.8
	90	50	52.7	53.4	53.0	51.8
						
	10	10	6.33	7.65	5.97	5.37
fenamiphos	50	60	29.2	28.1	25.6	33.8
	90	200	74.4	86.2	68.7	68.3
						
	10	10	10.3	8.5	11.3	11
mefloquine	50	250	240	205	248	267
	90	500	492	530	477	470

### Exposures

L1 larvae (2.5 × 10^5^) were suspended in T-75 flasks containing 30 mL CeHR medium and grown until 90% of the population had developed to the mid-vulval L4 larval stage–two flasks were allotted for each condition to provide adequate biomass for RNA and protein preparation. The worms were treated with mefloquine (Ash Stevens, Inc., Detroit, MI), dichlorvos, or fenamiphos (Chem Service, Inc., West Chester, PA) for 8 h or allowed to develop as a control; a sample was taken for chemical analysis to verify exposure concentration (Table 1). Each exposure was repeated three times.

Worms were harvested by centrifugation (800 × g for 3 min at 4°C), and the supernatant was aspirated. Samples for protein extraction were washed once with 0.1 M NaCl, centrifuged (800 × g for 3 min at 4°C), and the supernatant was aspirated. The pellets for protein and RNA extraction were suspended in the residual liquid, flash frozen by drop-wise addition to liquid nitrogen, and stored at -80°C.

### Chemical analysis

Chemicals were analyzed on a Hewlett-Packard Model 6890 Gas Chromatograph equipped with a 6890 model series auto injector. Ions were measured for fenamiphos with a 5973 Mass Selective Detector, for mefloquine with a flame ionization detector, and for dichlorvos with an electron capture detector. Analytical standards were purchased from Chem Service, Inc.

### RNA methods

#### Extraction and labeling

Frozen worm droplets were pulverized in liquid N_2 _using a pre-chilled mortar and pestle. The pulverized worms were transferred to 6 mL Trizol (Invitrogen, Carlsbad, CA) and homogenized in a dounce homogenizer. RNA was purified according to the manufacturer's protocol and precipitated with isopropyl alcohol. After centrifugation, the RNA pellet was dried, dissolved in water, and subjected to an additional round of purification using the RNeasy Maxi Kit (Qiagen, Valencia, CA) according to the manufacturer's directions. The quality and yield of the preparation was assessed throughout processing and labeling using a 2100 Bioanalyzer (Agilent, Santa Clara, CA), and when necessary, the mass yield was confirmed using an ND-1000 spectrophotometer (Nanodrop Technologies, Wilmington, DE).

Poly(A)+ RNA was isolated from the total RNA using OligoTex (Qiagen) essentially as described by the manufacturer. Two micrograms of poly(A)+ RNA (adjusted for rRNA contamination) was used as the template for cDNA synthesis using the SuperScript Choice Kit (Invitrogen) per the manufacturer's recommendations except that (1) a high pressure liquid chromatography (HPLC)-purified T_24_T7 promoter primer (Integrated DNA Technologies, Coralville, IA) was used to initiate first strand synthesis; (2) the second strand synthesis was not terminated using EDTA since we found that EDTA carryover interfered with subsequent enzymatic manipulations; and (3) PelletPaint (Novagen, Madison, WI) was used in place of glycogen for precipitation. Biotin labeled cRNA was synthesized from the T7 promoter incorporated in the cDNA using the BioArray High Yield RNA Transcript Labeling Kit (Enzo Life Sciences, Farmingdale, NY) per the manufacturer's recommendations; approximately 1 μg of cDNA was used for synthesis. cRNA was purified from unincorporated nucleotides and other reaction components using the RNeasy Mini Kit (Qiagen).

#### Microarrays

cRNA samples were hybridized to *C. elegans *whole genome GeneChips (Affymetrix, Santa Clara, CA), processed, and scanned at the Walter Reed Army Institute of Research Vaccine Genomics Laboratory, Rockville, MD using Affymetrix instrumentation and with hybridization, washing, and scanning parameters provided by the manufacturer [40].

#### Microarray data analysis

Microarray data was processed using the robust multi-array averaging method (RMA) [41]. To verify inter-replicate reproducibility, replicate samples were subjected to pairwise correlation analysis of all probe sets. For the vast majority of replicate pairs, the R^2 ^value was greater than or equal to 0.95, and no replicates were included with R^2 ^< 0.92. A Present, Absent, or Marginal call for each probe set was determined using the R statistical package [42] and the Bioconductor [43] implementation of the Affymetrix MAS 5.0 algorithm (affy package 1.12.2). Only probe sets with at least three present calls in the complete data set were retained for further analysis. This procedure removed 5,623 out of the total 22,624 probe sets on the microarray from the analysis. We have observed that even when a multiple test correction is used in ANOVA with high dimensional microarray data, small differences in gene expression that are not credible on careful inspection of the signal intensities can be assigned highly significant *p *values. To reduce the impact of this problem, we retained a final tally of 4,999 probe sets that passed the Present/Absent screen and changed by at least 1.8 fold from control for statistical analyses.

#### Support vector machine for dosing standardization

**Figure 1:**
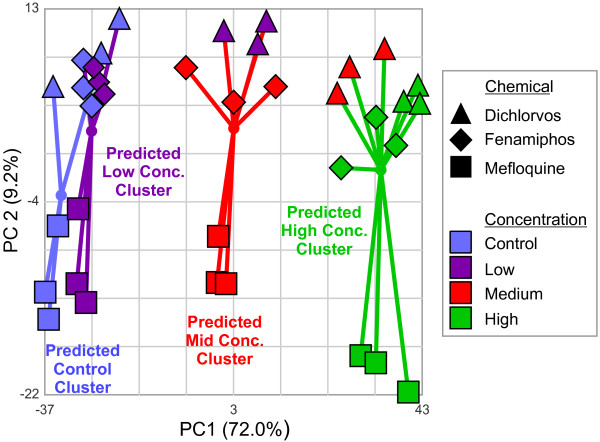
**Clustering of samples of worms exposed to standardized concentrations of dichlorvos, fenamiphos, and mefloquine**. Principal components analysis plot depicting the clustering of samples of worms exposed to standardized concentrations of the three toxicants based on gene expression levels. Three replicates are shown. Nominal concentration classes are indicated in color, and chemical exposure groups (including respective controls) are indicated by shapes. Low, medium, and high concentrations refer to EC_10_, EC_50_, and EC_90 _from the developmental inhibition assay. A support vector machine (SVM) trained on a data set lacking dichlorvos exposed worms was used to classify the samples based on gene expression levels. Samples which the SVM predicts to have the same concentration level are joined by lines to a centroid. The percent variance in the data explained by each principal component is shown in parentheses.

On inspection, the standardized concentrations of dichlorvos seemed to exert relatively greater effects on the patterns of gene expression in exposed worms than mefloquine or fenamiphos, yielding a right shifted pattern of gene expression (see Figure 1). To confirm this observation, we used a support vector machine (SVM, [44] Partek Pro Genomics Suite 6.0–default settings) to predict an apparent concentration (control, low, mid, or high) for each chemical to which the worms had been exposed based on patterns of gene expression. For SVM modeling, we used data from worms exposed to cadmium and acrylamide in parallel experiments (unpublished data) in addition to fenamiphos and mefloquine. No dichlorvos data were included. To take the differences between the measured and targeted concentrations of chemicals (Table 1 and not shown) into account for this analysis, we calculated an adjusted measure of developmental arrest by prorating the target level of arrest (10%, 50% or 90%) by the ratio of the measured concentration of toxicant to the target concentration (Equation 1). The 100 probe sets with the highest partial correlations to this adjusted value were used to train the SVM.

(1)

#### Identification of OP specific gene changes

For identification of OP-specific gene changes, we removed the fenamiphos low concentration and the dichlorvos high concentration data from consideration. The samples for the two remaining exposed concentrations for each OP were grouped based on the SVM classification as either "mid concentration OP" or "high concentration OP." The OP control samples and all of the mefloquine samples were placed into a third "no OP" class. An ANOVA identified 500 probe sets that are significantly different (FDR ≤ 10^-4^; false discovery rate, [45]) among the three classes. To eliminate those genes whose expression was even marginally affected by mefloquine exposure, we next removed probe sets that changed by 1.5 fold or more from control at any concentration, in any replicate of the mefloquine data, to generate a list of 94 probe sets differentially affected by OP exposure, but unaffected by mefloquine.

Following statistical identification of the 94 differentially expressed probe sets, we inspected their mapping on the *C. elegans *genome (based on WormBase oligo mapping; WormBase release 180) [46] and found 20 of them that represent genes with at least one additional probe set on the microarray that was not identified, based on our strict criteria, as a probe set specifically affected by OP exposure. In most cases, these probe sets have similar patterns of expression but display slight differences in the magnitude of the fold change from control with the result that one probe set passed the fold change or statistical cut off while the other(s) did not. In two instances, the selected and rejected probe sets targeted different splice variants of the same gene. In another, the probe set showed a response to mefloquine, but the change was in the opposite direction compared to the OP responses; we deemed this to be an OP specific gene change. In a final case, the unidentified probe set had a signal intensity below background (indicated by no Present calls). We retained all 15 of these probe sets.

**Figure 2:**
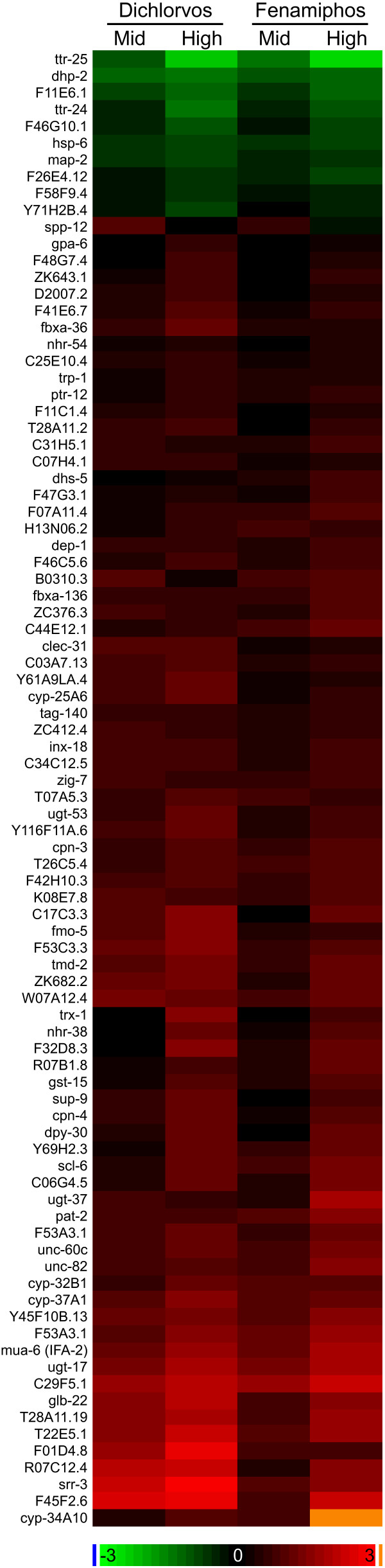
**Changes in expression levels of genes specifically affected by OP exposure**. Heatmap depicting the average changes in expression levels of genes affected by OP exposure. Gene or sequence names are shown at the left of the heatmap. The color bar indicates log_2 _differences from the control for each chemical. Concentrations are based on SVM predictions.

However, we excluded probe sets for five genes each recognized by two probe sets. For four of these genes, one but not the other of the probe sets showed changes in expression upon mefloquine exposure with no readily apparent explanation for the differences. The other one hybridized to two genes, and we could not resolve which gene was being measured. One final probe set was removed because it was called Present (by MAS 5.0 algorithm) in only two of the OP exposed samples; the third sample in which it was called Present was a mefloquine sample. After these adjustments, a group of 88 probe sets (representing 87 genes) that respond to OP but not mefloquine exposure remained (Table 2, Figure 2).

**Table 2:** Genes specifically affected by organophosphorus pesticide exposure

**Probe Set**	**WBID^a^**	**Gene^b^**	**Description**	**Biological Role**	**Dic^c^**	**Fen^d^**
191443_at	WBGene00013078	*ttr-25*	unknown function – contains transthyretin-like family domain		-5.1	-5.6

192157_at	WBGene00000964	*dhp-2*	dihydropyrimidinase		-2.7	-2.4

187663_at	WBGene00008706	F11E6.1	glucocerebrosidase	Lipid metabolism	-2.4	-2.3

173412_s_at	WBGene00013077	*ttr-24*	unknown function – contains transthyretin-like family domain		-2.5	-1.9

181137_at	WBGene00009796	F46G10.1	unknown function – contains potassium channel tetramerization domain		-2.2	-1.7

192644_at	WBGene00002010	*hsp-6*	heat shock 70 protein	Stress	-1.8	-1.8

184978_at	WBGene00003130	*map-2*	methionine aminopeptidase	Anti-apoptosis	-1.8	-1.6

191082_at	WBGene00009165	F26E4.12	glutathione peroxidase	Redox	-1.6	-1.9

183671_at	WBGene00019058	F58F9.4	unknown function – contains DUF272 domain		-1.7	-1.5

185811_at	WBGene00022194	Y71H2B.4	unknown function		-1.7	-1.4

178599_at	WBGene00004997	*spp-12*	saposin like protein family	Lipid metabolism	-1.1	-1.2

193531_at	WBGene00001668	*gpa-6*	G protein alpha subunit involved in chemosensation	Sensory	1.7	1.3

183021_at	WBGene00018615	F48G7.4	unknown function		1.9	1.3

186977_at	WBGene00014033	ZK643.1	arrestin like protein	Sensory	1.9	1.6

175989_at	WBGene00017042	D2007.2	unknown function – contains MSP domain	Axon Guidance	1.9	1.5

180587_at	WBGene00018288	F41E6.7	unknown function		2.0	1.7

176365_at	WBGene00022479	*fbxa-36*	F-box A protein	Ubiquitination	2.4	1.4

193559_s_at	WBGene00003644	*nhr-54*	nuclear hormone receptor-54	Transcription factor	1.3	1.5

185495_at	WBGene00016094	C25E10.4	predicted transporter/transmembrane protein	Membrane channel	1.6	1.4

193861_at	WBGene00006614	*trp-1*	transient receptor potential ion channel involved in sensory transduction	Sensory, membrane channel	1.5	1.5

188133_at	WBGene00004226	*ptr-12*	patch related family	Sterol trafficking	1.7	1.7

179248_at	WBGene00008693	F11C1.4	unknown function		1.6	1.4

184849_at	WBGene00020869	T28A11.2	unknown function – contains DUF19 domains		1.8	1.5

179132_at	WBGene00007854	C31H5.1	alpha/beta hydrolase of unknown function	Sensory	1.4	1.8

178276_at	WBGene00007421	C07H4.1	unknown function		1.7	1.4

175147_at	WBGene00000969	*dhs-5*	dehydrogenase, short chain – possible steroid dehydrogenase	Detoxification	1.2	1.8

174374_s_at	WBGene00018576	F47G3.1	unknown function	Sensory	1.4	1.9

192917_s_at	WBGene00008547	F07A11.4	ubiquitin carboxyl-terminal hydrolase	Ubiquitination	1.6	2.1

192646_at	WBGene00010396	H13N06.2	unknown function – contains VWA invertebrate integrin domain		1.5	1.5

191570_s_at	WBGene00009717	*dep-1*	class III receptor protein tyrosine phosphatase (R-PTP)	Signaling	1.6	1.8

193155_s_at	WBGene00009781	F46C5.6	unknown function – contains 2 HEAT repeat domains		1.8	1.9

187505_at	WBGene00015139	B0310.3	unknown function		1.2	2.0

179197_s_at	WBGene00007672	*fbxa-136*	F-box A protein	Ubiquitination	1.6	2.1

189341_s_at	WBGene00013875	ZC376.3	b-type carboxylesterase	Detoxification	1.7	2.1

185117_at	WBGene00016657	C44E12.1	N-acyl-L-amino-acid amidohydrolase	Amino acid metabolism	1.7	2.5

193763_at	WBGene00009854	*clec-31*	unknown function – contains 2 C-type lectin domains		2.1	1.5

186028_at	WBGene00015371	C03A7.13	UDP-glucuronosyl/glucosyl transferase	Detoxification	2.1	1.6

177226_s_at	WBGene00022016	Y61A9LA.4	unknown function		2.4	1.5

189292_at	WBGene00019438	*cyp-25A6*	cytochrome P450	Detoxification	2.4	1.7

191150_at	WBGene00006486	*tag-140*	predicted Zn transporter	Membrane channel	1.7	1.7

178686_s_at	WBGene00013885	ZC412.4	unknown function		1.7	1.6

181726_at	WBGene00002140	*inx-18*	innexin – invertebrate gap junction protein	Membrane channel	1.8	1.8

188880_at	WBGene00007924	C34C12.5	Ras suppressor protein	Cell Adhesion	1.9	1.8

188007_at	WBGene00006984	*zig-7*	2(Zwei) IG-domain protein	Cell Adhesion	1.7	1.8

193031_at	WBGene00011556	T07A5.3	sugar phosphate permease	Membrane channel	2.1	1.5

191623_at	WBGene00020182	*ugt-53*	UDP-glucuronosyl/glucosyl transferase	Detoxification	2.3	1.8

186581_at	WBGene00013819	Y116F11A.6	unknown function – contains caspase recruitment domain	Apoptosis/Cell Death	2.5	1.7

188459_s_at	WBGene00000779	*cpn-3*	Calponin	Actin related	2.0	2.1

177598_at	WBGene00012034	T26C5.4	unknown function		2.1	2.1

176229_at	WBGene00018367	F42H10.3	unknown function – contains Nebulin repeat domain	Actin related, Cell Adhesion	2.1	2.0

193302_at	WBGene00010675	K08E7.8	unknown function – contain leucine rich repeat domain	Apoptosis/Cell Death	2.0	2.1

183894_at	WBGene00015889	C17C3.3	acyl co-A thioesterase	Lipid metabolism	3.1	2.4

189169_s_at	WBGene00001480	*fmo-5*	flavin-containing monooxygenase	Detoxification	3.1	1.6

175285_at	WBGene00018747	F53C3.3	unknown function – contains CX module		3.3	2.1

188099_s_at	WBGene00006582	*tmd-2*	Tropomodulin	Actin related	2.8	2.4

173824_at	WBGene00022788	ZK682.2	sugar phosphate permease	Membrane channel	2.7	2.5

190692_at	WBGene00012322	W07A12.4	unknown function – contains btb/poz domain	Ubiquitination	2.4	2.3

189557_at	WBGene00015062	*trx-1*	Thioredoxin	Redox	3.2	1.7

193582_at	WBGene00003629	*nhr-38*	nuclear hormone receptor involved in thermosensation	Sensory	2.4	2.1

178886_at	WBGene00009328	F32D8.3	unknown function – contains trypsin Inhibitor like cysteine rich domain		3.1	2.4

190432_at	WBGene00001608	R07B1.8	unknown function – contains GTP binding domain		1.9	2.2

190957_at	WBGene00001763	*gst-15*	glutathione S-transferase	Redox	2.2	2.1

188131_s_at	WBGene00006318	*sup-9*	TWK potassium channel	Membrane channel	2.4	1.9

184319_at	WBGene00000780	*cpn-4*	Calponin	Actin related	2.5	2.1

190211_at	WBGene00008746	*dpy-30*	adenylate kinase like		2.5	2.5

177839_s_at	WBGene00013481	Y69H2.3	unknown function – contains trypsin inhibitor like cysteine rich domain		2.3	2.3

192432_at	WBGene00008028	*scl-6*	defense-related protein containing SCP domain		2.4	2.7

191916_at	WBGene00015559	C06G4.5	G-protein coupled receptor		2.5	2.6

190719_at	WBGene00017332	*ugt-37*	UDP-glucuronosyl/glucosyl transferase	Detoxification	1.6	4.2

192997_s_at	WBGene00018832	*pat-2*	vitronectin receptor, alpha subunit	Actin related, Cell Adhesion	1.8	3.0

172944_s_at	WBGene00018720	F53A3.1	unknown function		2.9	3.7

193210_s_at	WBGene00006794	*unc-60c*	actin depolymerizing factor	Actin related	2.5	2.6

187747_at	WBGene00006814	*unc-82*	serine/threonine protein kinase required for thick filament organization		2.2	2.9

173480_s_at	WBGene00021167	*cyp-32B1*	cytochrome P450	Detoxification	2.5	2.2

173698_s_at	WBGene00008499	*cyp-37A1*	cytochrome P450	Detoxification	3.0	2.3

186562_s_at	WBGene00012875	Y45F10B.13	unknown function		2.8	3.3

179847_s_at	WBGene00018720	F53A3.1	unknown function		2.3	2.5

193108_s_at	WBGene00003485	*mua-6 *(*ifa-2*)	intermediate filament protein	Cell Adhesion	3.7	3.9

191689_s_at	WBGene00007422	*ugt-17*	UDP-glucuronosyl/glucosyl transferase	Detoxification	4.1	4.3

183314_at	WBGene00016210	C29F5.1	unknown function		4.7	5.5

178198_at	WBGene00011251	*glb-22*	unknown function – contains globin domain		4.7	3.3

186720_at	WBGene00020881	T28A11.19	predicted secreted cysteine rich protein		4.3	3.7

183178_at	WBGene00020690	T22E5.1	unknown function	Actin related	5.2	3.5

190445_at	WBGene00008490	F01D4.8	cysteine synthase/cystathionine beta-synthase family	Redox	6.6	1.8

185116_s_at	WBGene00019934	R07C12.4	unknown function		5.5	3.1

177733_at	WBGene00005654	*srr-3*	serpentine receptor, class R	Sensory	7.5	2.9

181201_at	WBGene00018479	F45F2.6	otopetrin like transmembrane protein	Sensory	6.7	5.1

189633_at	WBGene00015045	*cyp-34A10*	cytochrome P450	Detoxification	2.2	21.2

a- Gene names and descriptions were derived from WormBase, release WS189 [46].

b- WormBase gene identifier.

c- Gene expression fold change values for dichlorvos SVM predicted high concentration

d- Gene expression fold change values for fenamiphos high concentration

#### Gene ontology analysis

In order to assist in interpreting the microarray data, DAVID [47-49] and GoMiner [50,51] were used to assess whether particular gene ontology terms occurred more frequently than expected by chance in the set of genes specifically affected by OP exposure. DAVID was run using the high stringency setting and the following annotation groups: Molecular Function level 4–5, Cellular Component level 4–5, Biological Function level 4–5, InterPro terms, and PIR keywords. Of the 88 probe sets submitted all but 9 were clustered by annotation. GoMiner was run through the web interface with default settings except that all ontology terms were used. The group of 88 probe sets that are specifically affected by OP exposure was compared to the annotation of the entire *C. elegans *genome for both DAVID and GoMiner for statistical evaluation. For DAVID analysis, we report the negative antilog of the Group Enrichment Score as *p *values.

#### Identification of OP discriminating gene changes

For identification of gene changes that discriminate between the OP exposures, only data from OP exposed samples and their respective controls were included. As above, SVM predicted concentrations were used for classification and the low fenamiphos and highest dichlorvos exposures were omitted. A 2-way ANOVA using the SVM predicted concentrations and exposure chemical (fenamiphos, dichlorvos, and control) revealed 28 probe sets with significantly different expression between the two OPs (FDR ≤ 10^-4^). Two of these genes are also targeted by additional probe sets which do not meet the fold difference criterion but are similar in expression pattern to the originally identified probe sets, so both original probe sets were retained. This list was further refined to include only probe sets which changed by at least 1.8 fold as a result of the exposure and between chemicals resulting in a final list of 24 probe sets, representing 23 genes.

Microarray data have been deposited in the Gene Expression Omnibus [52], Accession Number GSE12298.

### Protein Methods

Complete details of sample processing, mass spectrometry, and data analysis may be found in Additional File 1: ProteinMethods.pdf. A brief description follows.

#### Purification and processing

Frozen worm droplets from the highest concentrations of fenamiphos and dichlorvos exposures and the unexposed controls were ground in liquid N_2 _and resuspended [40 mM Tris, 1 mM EGTA, and 1 × Protease Inhibitor Cocktail (Sigma-Aldrich, St. Louis, MO)]. The suspension was sonicated, clarified by centrifugation, and lyophilized. Four milligrams of protein from each sample were denatured in 8 M UREA and dithiothreitol and then acetylated with iodoacetamide. After dilution to 1 M urea, the samples were digested with trypsin (Promega, Madison, WI).

#### Peptide analysis

The digested peptides were desalted, dried under vacuum, reconstituted in 10% acetonitrile, and fractionated using mixed mode ion chromatography with a Polycat A column and Polywax LP column in series (PolyLC Inc., Columbia, MD). Eight time based fractions were collected. Each fraction was analyzed using a nanoACQUITY UPLC coupled to a QTOF Premier quadrupole, orthogonal acceleration time-of-flight tandem mass spectrometer (Waters, Milford, MA). Data were collected over the 50–1990 mass to charge (m/z) range using the Waters Protein Expression MS^E ^method, which alternates between low energy scans to survey the precursor ions and high collision energy scans to fragment all of the precursor ions. Computational methods are used to assign fragment ions to precursor ions based on elution profiles [53,54].

#### Proteomic data analysis

Mass spectrometry data were processed using Protein Lynx Global Server (PLGS) version 2.3 (build 23) with Expression version 2 (Waters). Data preparation and workflow parameters were set to manufacturer's default with the exception of a 785.8426 lock mass, allowing deamidated asparagine and glutamine and oxidated methionine as variable modifications, and enabling PPM calc. The protein identification database contained all *C. elegans *RefSeq sequences (download date August 8, 2007) [55] and likely contaminant proteins including bovine serum albumin, human keratins, and porcine trypsin.

For our investigation of proteins that change in abundance upon OP exposure, we combined the high concentrations data sets for dichlorvos and fenamiphos into one group and compared it to the combined unexposed controls for these exposures. We have only reported proteins that were identified in at least four replicates of the condition where the protein is at the higher abundance. Those present in both conditions and changing by 1.5 fold we consider as quantitative changes, and those absent in the other condition as experiencing qualitative changes in abundance.

## Results and Discussion

To investigate the effects of OP AChE inhibitors on global gene and protein expression, we exposed synchronized cultures of *C.elegans *to standardized concentrations of the three neurotoxicants, fenamiphos, dichlorvos, and mefloquine. We determined the percentage of worms that failed to develop from mid-vulval L4 larvae to early gravid adult (EGA) during a 24-hour exposure in range finding experiments and set benchmark concentrations for 10%, 50%, and 90% developmental inhibition. In control cultures, 100% of the worms developed to EGA. Synchronized cultures of *C. elegans *at the mid-vulval L4 stage were exposed to the indicated concentration of toxicant (Table 1) for 8 h. Unexposed cultures served as controls. Protein and mRNA isolated from the exposed and unexposed control nematodes were analyzed by mass spectrometry or whole genome microarray, respectively. In general, the worms exposed to the OPs appeared to have limited mobility and suffered from hypercontraction of their muscles. In feeding studies, the exposed worms displayed at least a minimal pharyngeal reflex (data not shown). However, it is unlikely that they fed normally, and we observed changes in gene and protein expression that are likely due to nutritional restriction (see below).

### Developmental genes

Because the dosing for these experiments was standardized based on the inhibition of developmental processes, we were concerned that the ensemble of probe sets we observed to change in response to OP exposure might be skewed toward genes involved in development. To address this issue, we compared two lists of genes. The first list contained developmentally regulated genes derived from an unpublished data set spanning the same developmental period as this experiment; the second list contained genes from the study at hand whose expression level is highly correlated with developmental inhibition for all three toxicants. Both sets comprised the 2000 probe sets with the lowest *p *values for the relevant desideratum. Only 438 probe sets are shared by the two lists. Furthermore, only 4 of the 88 probe sets affected by OP but not mefloquine exposure (see below) appear in the developmental data set. We concluded that our experimental design effectively excluded developmentally regulated genes.

### Cross chemical standardization

Preliminary examination of the data suggested the expression levels of genes in worms exposed to the three standardized concentration levels of dichlorvos were shifted toward those seen in worms exposed to higher standard concentrations of mefloquine and fenamiphos. Figure 1 presents the results of a principal components analysis (PCA) performed on 1110 probe sets that are statistically different by concentration (2-way ANOVA, concentration and exposure group; FDR ≤ 10^-5^). It is noteworthy that the dichlorvos low concentration samples cluster with the fenamiphos and mefloquine mid concentration samples, and the dichlorvos mid and high concentration samples cluster with the fenamiphos and mefloquine high concentration samples. Because this shift in gene expression cannot be fully accounted for by deviations in the administered concentrations from the nominal concentrations, we verified the apparent clustering by assigning predicted concentration levels to all the samples with a support vector machine (SVM). The SVM was trained on gene expression levels from a data set that contains no dichlorvos exposure data but does include data from mefloquine, fenamiphos, and two additional toxicant exposures (see Methods). The SVM classification results support our conclusion that the dichlorvos effects are shifted toward higher concentration levels with respect to mefloquine and fenamiphos. For all subsequent analysis of microarray data, we used the predicted rather than nominal concentration levels for dichlorvos.

### OP specific responses

In order to identify genes that are regulated by exposure to OPs but not by generalized stress caused by toxic chemical exposure, we compared the expression of genes in worms exposed to two OPs, dichlorvos and fenamiphos, to gene expression in worms exposed to the unrelated toxicant mefloquine and unexposed controls. Eighty-eight probe sets representing 87 genes respond specifically to OP intoxication (Table 2 and Figure 2). The changes in the expression of this set of genes represent responses by the worm that are specific to OP exposure and are not a result of generalized stress or developmental delays, as they do not respond to the mefloquine exposures. In a separate analysis of proteins whose abundance was affected by OP exposure, we found 34 proteins whose level of expression changed in response to dichlorvos and fenamiphos intoxication (Table 3). While the differences in the expression of some of these proteins might result from generalized stress responses or from developmental delays (no mefloquine out-group was included in the proteomic analysis), many appear to be part of the same biological processes involving the OP specific gene set. These biological processes included muscle damage, cell death, and detoxification.

**Table 3:** Proteomic changes upon organophosphorus pesticide intoxication

						**Genomic Results^f^**		
						
**Gene^a^**	**Proteins^b^**	**Description**	**Fold** **Change^c^**	**Quant Fracts^d^**	**Total Fracts^e^**	**MH**	**DH**	**FH**
C29F5.1	NP_495267.1	unknown function	OP only	0	1	-1.2	5.6	5.5

C42D4.1	NP_501136.1	unknown function – predicted alpha-helical protein	OP only	0	2	14.8	5.4	6.7

C42D4.3	NP_501132.1	unknown function – contains fibronectin domain	OP only	0	2	4.9	4.3	5.0

*cul-3*	NP_503151.1	Cullin	OP only	0	1	-1.3	-1.4	-1.5

E04F6.5	NP_001022062.1 NP_001022063.1	Very-long-chain acyl-CoA dehydrogenase	OP only	0	1	1.1	1.0	-1.2

K03E5.2	NP_001021535.1 NP_001021536.1 NP_001040674.1	predicted calponin	OP only	0	2	-1.2	3.0	2.9

T28F4.5	NP_492102.1	homolog of Death Associated Protein 1	OP only	0	1	2.6	1.7	1.4

Y57G11A.3	NP_502756.1	unknown function – contains LIM domain	OP only	1	1	1.6	4.4	6.0

*gei-7*	NP_001021367.1 NP_503306.1	isocitrate lyase/malate synthase	3.0	4	4	3.3	4.5	3.8

C32D5.8	NP_001022003.1 NP_001022004.1	unknown function – contains thioredoxin domain	2.9	1	1	2.7	4.3	3.9

T19B10.2	NP_505848.1	unknown function	2.3	5	6	9.3	4.3	4.8

C06A8.3	NP_495640.1	homolog of OV-17 hypodermal antigen	2.2	4	6	2.4	2.1	2.2

*nex-1*	NP_498109.1	Annexin	2.2	3	4	2.2	2.8	2.3

*ifb-1*	NP_495136.1 NP_495137.1	intermediate filament, B	1.9	3	4	1.1	1.5	1.5

*sap-1*	NP_494763.1	U2-associated snRNP A' protein	1.8	1	1	-1.3	-1.3	-1.2

ZK909.3	NP_493608.2	guanosine polyphosphate pyrophosphohydrolase/synthase	1.8	1	1	3.7	2.5	2.5

*pfn-3*	NP_508205.1	Profiling	1.7	2	2	1.2	3.8	4.3

H34C03.2	NP_501035.1	ubiquitin C-terminal hydrolase	1.7	1	1	-1.2	-1.4	-1.3

*spp-14*	NP_001041271.1	saposin like protein family	1.6	1	1			

*unc-60c*	NP_503427.2	cofilin – actin depolymerizing factor	1.6	2	2	1.9	1.8	1.8

*tag-273a*	NP_001023516.1	unknown function – contains LIM domain	1.5	1	1	-1.2	2.0	3.5

*unc-87*	NP_001021092.1 NP_001021093.1 NP_001021094.1	myofilament associated protein	1.5	4	6	-2.2	1.2	1.6

F22F7.1	NP_503577.1 NP_872194.1	uncharacterized membrane protein	-1.6	1	2	-2.5	-4.3	-4.4

*ttr-16*	NP_502060.1	transthyretin like family	-1.6	1	1	1.1	-1.0	1.0

*dct-16*	NP_507944.1	unknown function – daf-16 regulated	-1.7	2	4	-4.6	-4.8	-5.1

*dsc-4*	NP_499903.3	microsomal triglyceride transfer protein, large subunit	-1.7	1	3	-1.3	-2.4	-2.0

*pmt-1*	NP_494990.2 NP_494991.1 NP_871997.1	phosphoethanolamine N-methyltransferase	-1.7	2	3	1.9	1.5	1.3

ZK1127.10	NP_495449.1	cystathionine gamma-lyase	-1.8	1	3	-1.3	-1.7	-1.9

*asp-1*	NP_741677.1	aspartyl protease	-1.9	3	4	1.2	-1.3	-1.2

*pod-2*	NP_001022400.1 NP_001022401.1	acetyl-CoA carboxylase domain	-2.0	1	6	2.2	1.0	-1.2

*asp-5*	NP_505135.1	aspartyl protease	-2.2	2	2	-1.0	-1.3	-1.1

*dct-18*	NP_496755.1	unknown function – daf-16 regulated	-2.4	3	3	-2.2	-1.9	-1.9

F48E3.3	NP_509268.1	UDP-glucose:glycoprotein glucosyltransferase domain	Cont only	0	2	1.2	-1.2	-1.0

*ifg-1*	NP_001022259.1 NP_001022260.1	initiation factor 4G	Cont only	0	2	1.4	1.0	1.1

a- Gene names and descriptions were derived from WormBase, release WS189 [46].

b- NCBI protein accession number

c- Protein fold change values for OP exposure. Proteins identified as "OP only" or "Cont only" were identified in at least 4 replicates of the OP-exposed or control samples and in no replicates of the other condition.

d- Number of fractions in which quantitative comparisons were made

e- Number of fractions in which protein was identified

f- Gene expression fold change values for mefloquine (MH), dichlorvos (DH), and fenamiphos (FH) high concentration exposure. Gene expression levels for H34C03.2 were below signal to noise threshold.

#### Muscle damage

In the lists of genes and proteins specifically affected by exposure to the OPs, we observed increases in the expression of a number of molecules involved with muscle structure and function, including genes encoding an intermediate filament, *ifa-2 *[56]; a *ras *suppressor, C34C12.5 [57]; a vitronectin receptor, *pat-2 *[58]; a cell adhesion molecule from the immunoglobulin superfamily, *zig-7 *[59]; the nematode homolog to actin regulator, LASP-1 (F42H10.3) [60]; and a serine/threonine protein kinase important for proper striated muscle structure and, perhaps, body wall attachment, *unc-82 *[61]. We also observed increases in expression of the IFB-1 protein which is co-expressed with the intermediate filament protein IFA-2 (see above) [62]. All of these genes and proteins are involved in cell adhesion, muscle attachment or structure, suggesting that muscle repair/regeneration responses may have occurred as a result of mechanical damage resulting from muscle hypercontraction. Interestingly, inhibiting synthesis of the *zig-7 *product with RNAi confers resistance to aldicarb, an AChE-inhibiting carbamate [63].

In addition, a number of transcripts and proteins modulating actin polymerization are also up-regulated, although these molecules are not necessarily muscle-specific. The expression of *unc-60*, a cofilin-like actin depolymerization factor, increases in both the proteomic and genomic assays. The expression of profilin (PFN-3), calponin genes (*cpn-3 *and *cpn-4*), and the K03E5.2 gene product, which contains a calponin repeat, is also induced. Calponins may play a role in regulation of myosin ATPase activity and muscle contraction [64]. Finally, the expression of the gene encoding the actin end cap and nebulin-binding protein, tropomodulin (*tmd-2*) is increased as is F42H10.3, a poorly described gene encoding a nebulin repeat domain.

Taken together, the data argue for an increased requirement for molecules involved in cytoskeletal and muscle structure and suggest ongoing cytoskeletal rearrangement and perhaps repair of the muscular system as a result of OP exposure, a conclusion that is consistent with our previous observation of convulsions in worms exposed to dichlorvos [37].

#### Cell death

We also found alterations in the expression of a number of genes and proteins involved in cell death. Neuronal death in response to OP exposure in *C. elegans *is consistent with the neurodegenerative effects of a gain of function mutation of *deg-3*, which encodes the nicotinic acetylcholine receptor (nAChR) [65], and with the occurrence of neuronal death in mammals in response to OP exposure [2]. We observed increased levels of the NEX-1 protein, which mediates apoptotic engulfment, and the *map-2 *metalloprotease gene was down-regulated; its human homolog is anti-apoptotic [66]. A possible additional indication of apoptotic activity is an apparent change in sphingolipid metabolism in OP exposed worms. The sphingolipid metabolites, ceramide and sphingosine, are involved in apoptosis and growth arrest, while other metabolites, such as sphingosine 1-phosphate, are anti-apoptotic [67]. F11E6.1, a glucocerebrosidase encoding gene, is up-regulated, and the expression of *spp-12*, a gene encoding a saposin-like protein which may be involved in sphingolipid metabolism, is altered (see below). However, these changes in lipid metabolism could also be responses to starvation or to disruption in the level of free acetylcholine.

At face value, the evidence argues against the occurrence of necrosis. *C. elegans *has six aspartyl protease genes (*asp-1 *through *asp-6*) which are believed to be under the control of the *daf-2*/insulin/IGF-1 regulatory pathway (see below) [68]. The *asp-3 *and *asp-4 *(and possibly *asp-1*) genes have been implicated in neuronal necrosis in RNAi experiments [69], and ASP-1 is required for necrotic cell death [70]. When we examined the expression of the aspartyl protease genes and proteins, we observed that the abundance of ASP-1 and ASP-5 proteins was reduced in worms exposed to OPs, although there was at most a marginal reduction in their transcript levels (average difference < 1.3 fold). In addition, the *asp-4 *transcript was down-regulated nearly three fold upon OP exposure. The reduction in aspartyl protease levels suggested by these observations is consistent with the known diminution of aspartyl protease activity during starvation [71], probably through auto-digestion. Intriguingly, starvation protects against neural degeneration [69], perhaps by reducing aspartyl protease activity.

At this point, it is uncertain to what extent cell death is occurring, and it is unclear how aspartyl protease activity is interacting with components of the cell death and starvation responses.

#### Detoxification

Many of the genes whose expression is induced in response to OP exposure appear to be involved in detoxification. Eight of the 87 genes up-regulated by OP exposure encode either cytochrome P450 monooxygenases or UDP-glucuronosyl/glucosyl transferases. Two additional induced genes, *fmo-5 *and *dhs-5*, encode proteins (flavin-containing monooxygenase and a short chain dehydrogenase, respectively) that have previously been shown to respond to xenobiotic toxicants [24,72] and are most likely involved in detoxification. ZC376.3, which encodes a type B carboxylesterase, may also play a role in detoxification as a carboxylesterase from *Lucilia cuprina *has been shown to provide resistance to OP insecticides through hydrolysis of the phosphate [73]. However, as many carboxylesterases are inhibited by OPs [74], the up-regulation of ZC376.3 could also represent an off-target, positive feedback loop (see below).

It is also noteworthy that the expression of genes for a number of membrane channels is up-regulated in response to OP intoxication. While some of these channels may play other roles, it is likely that some of them are involved in detoxification. It is well known that the multidrug resistance gene (*mdr-1*) codes for a P-glycoprotein family ATP-dependent efflux transporter [75]. Furthermore, there are numerous examples in which detoxification includes the export of the toxicant from the cell [76].

When gene ontology analyses were performed using the 87 genes specifically affected by OP exposure, only categories containing genes plausibly involved in detoxification were identified as being enriched in the data set. GoMiner [50] identified one enriched gene ontology category (GO:0004497 monooxygenase activity; FDR = 0.032) containing four cytochrome P450 and one flavin-containing monooxygenase genes (*cyp-25A6, cyp-32B1, cyp-34A10, cyp-37A1, fmo-5*). Using DAVID [47,48], the same five monooxygenase genes were found in an annotation term cluster (*p *= 0.010). In addition, four known or putative UDP-glucuronosyl transferases (*ugt-17, ugt-37*, C03A7.13, and NM071370) were found in a second annotation term cluster (*p *= 0.033) supporting our suggestion above that the expression of genes involved in detoxification is altered in response to OP exposure.

Strikingly, the expression of the genes for these putative detoxification proteins is induced by OP exposure with some specificity since mefloquine fails to induce them; hence, there may be detoxification pathways specific to OPs and related chemicals.

#### DAF-16 *modulation*

The transactivator DAF-16 appears to be a key modulator of the changes in OP-specific gene and protein expression. Several signaling pathways converge directly on DAF-16 including the *daf-2*/insulin/IGF-1 pathway involved in stress and starvation, the PEP-2 innate immunity response pathway, the heat shock pathway, and other stress pathways mediated by jun kinase (JNK-1) and other mitogen-activated protein kinases (MAPKs). Responses to other stimuli appear to be transduced through DAF-16 by cofactor interaction [77,78] making DAF-16 a critical integrator of stress signals. We observed alterations in the expression of a number of genes and proteins under DAF-16 control including several aspartyl proteases (ASP-1, *asp-4*, and ASP-5; see above) [68] and molecules implicated in the fasting response (GEI-7 and ACS-11) [79] and in lipid transport and metabolism (*far-2*, C17C3.3, F11E6.1, *spp-12*) [46,80,81]. Two proteins, DCT-16 and DCT-18, downstream of DAF-16 [82] were found to be down-regulated. A curious observation is that the expression of the saposin gene, *spp-12*, which is known to be governed by DAF-16 [80], increases and then falls as the OP concentration rises. This gene appears to function as part of the innate immunity system and in sphingolipid metabolism. How DAF-16 interacts with the other physiological processes in play in these exposures is not at present clear. Nevertheless, as in the case of detoxification, the OP-induced DAF-16 mediated responses appear to be distinct from those elicited by mefloquine exposure.

#### Alternative targets

We were particularly interested in attempting to find pathways of OP intoxication and response that were not easily explainable as a direct result of inhibition of AChE. The worm homologs to neuropathy target esterase (NTE) were of particular interest because this enzyme is a known target for OP inhibition. The NTE protein affects lipid metabolism, and its inhibition causes axon damage [2]. There are two genes in the *C. elegans *genome homologous to the vertebrate secondary OP target, NTE (ZK370.4 and M110.7; [27] and unpublished observations). Expression of the ZK370.4 gene changed only slightly (1.5×) under any of the conditions tested; and the expression level of the other gene, M110.7, did not differ between fenamiphos exposed and control nematodes; however, expression was reduced in both the mefloquine and dichlorvos exposed animals in comparison with control (2.1 and 1.6 fold respectively in high concentration). Since the expression of M110.7 increases in control worms over the course of the experiment (unpublished data), it is not clear whether the decreased expression of the gene during mefloquine and dichlorvos exposure is an authentic toxic effect or whether it is simply the result of developmental inhibition. If the mefloquine and dichlorvos responses reflect a developmental effect, then fenamiphos exposure must be stimulating the expression of the gene in the developmentally retarded animals. The mechanism underlying such an effect is not clear, but as discussed below, dichlorvos and fenamiphos elicit different responses from a number of different genes.

We found several genes for enzymes with serine active sites that are specifically up-regulated upon OP exposure, perhaps as a result of a feedback loop since their activity could be inhibited directly by OPs. These include C17C3.3, C31H5.1, and ZC376.3; at present we cannot definitively ascribe functions to any of the products of these genes.

### Differential gene expression between OPs

**Figure 3:**
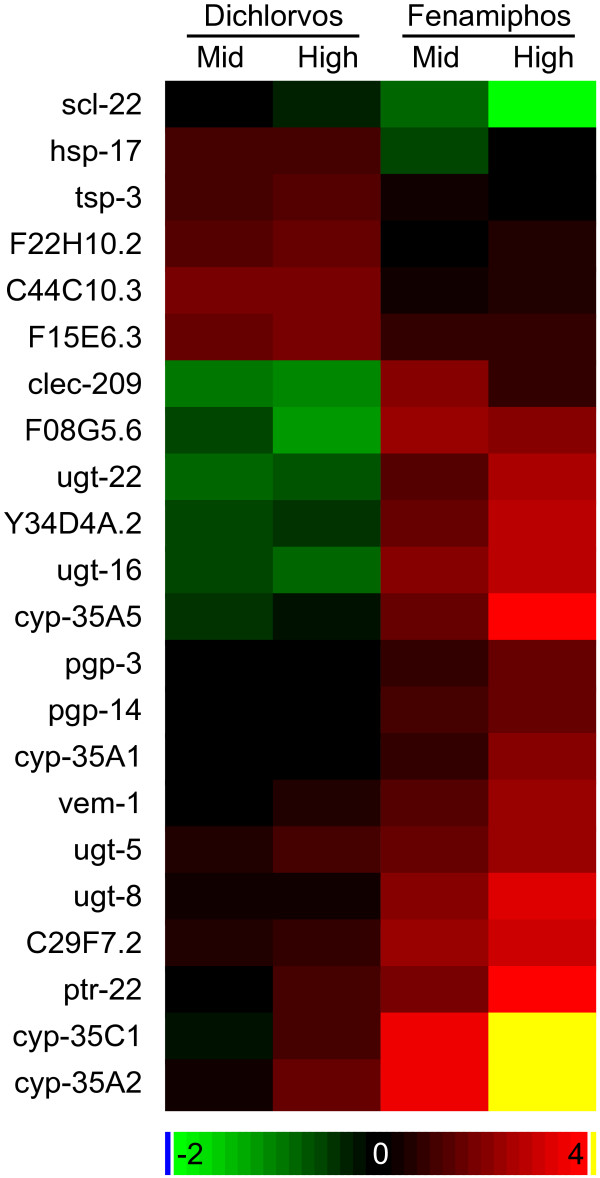
**Changes in expression levels of genes differentially affected by dichlorvos and fenamiphos exposure**. Heatmap depicting the average changes in expression levels of genes differentially affected by the exposure to the two OPs. Gene or sequence names are shown at the left of the heatmap. The color bar indicates log_2 _differences from the control for each chemical. Concentrations are based on SVM predictions.

In addition to finding genes that responded specifically to OPs, we wanted to identify genes that could discriminate between exposures to the two different OPs. We selected 23 probe sets, representing 22 different genes, showing a statistically significant difference between the two OPs and robust 1.8 fold difference between the control and exposed conditions (Table 4, Figure 3). Nearly half of these (9) encode phase I or phase II detoxification enzymes (UGT or cytochrome P450), and three encode P-glycoproteins (*pgp-3*, *pgp-14 *and C44C10.3), which are also involved in detoxification [83]. It is likely that the differences in gene expression reflect differences in the chemical structures of the compounds and the consequent activation of different detoxification pathways. Both molecules have two small hydrocarbon substituents, but dichlorvos is a phosphate ester with a dichlorovinyl group, and fenamiphos is a phosphoramidate with an aryl ring group [84].

**Table 4:** Genes differentially affected by dichlorvos and fenamiphos exposure

**ProbeSet**	**WBID^a^**	**Gene^b^**	**Description**	**Biological Role**	**Dic^c^**	**Fen^d^**
191857_s_at	WBGene00011462	*scl-22*	SCP-like extracellular protein		-1.2	-3.7

189735_at	WBGene00002021	*hsp-17*	heat shock protein	Stress	2.0	-1.0

190518_at	WBGene00006629	*tsp-3*	tetraspanin		2.5	1.0

184400_at	WBGene00017726	F22H10.2	unknown		3.2	1.7

192142_at	WBGene00008084	C44C10.3	major facilitator superfamily transporter	Detoxification	3.6	1.4

180425_s_at	WBGene00017484	F15E6.3	RRM-type RNA binding protein	miRNA regulation	4.0	1.9

180592_s_at	WBGene00021224	*clec-209*	unknown function – contains c-type lectin domain		-2.1	2.0

178843_at	WBGene00008584	F08G5.6	CUB domain containing protein		-2.3	4.9

193604_at	WBGene00007455	*ugt-22*	UDP-glucoronosyl and UDP-glucosyl transferases	Phase 2 enzyme	-1.6	6.7

183703_s_at	WBGene00012788	Y43D4A.2	UDP-glucoronosyl and UDP-glucosyl transferases	Phase 2 enzyme	-1.4	7.9

191418_at	WBGene00013901	*ugt-16*	UDP-glucoronosyl and UDP-glucosyl transferases	Phase 2 enzyme	-1.8	7.7

189394_at	WBGene00019473	*cyp-35A5*	cytochrome P450	Phase 1 enzyme	-1.1	15.9

193924_at	WBGene00003997	*pgp-3*	P-glycoprotein 3	Detoxification	1.0	3.1

190248_s_at	WBGene00004008	*pgp-14*	P-glycoprotein 14	Detoxification	-1.1	3.3

185525_at	WBGene00015399	*cyp-35A1*	cytochrome P450	Phase 1 enzyme	1.1	4.3

188031_s_at	WBGene00006890	*vem-1*	putative steroid membrane receptor – involved in axon guidance		1.6	5.7

191066_s_at	WBGene00013906	*ugt-5*	UDP-glucoronosyl and UDP-glucosyl transferases	Phase 2 enzyme	2.2	5.6

188962_at	WBGene00019234	*ugt-8*	ugt family, 7TM chemoreceptor	Phase 2 enzyme	1.2	10.4

178316_at	WBGene00007811	C29F7.2	unknown		2.0	9.8

188217_at	WBGene00004236	*ptr-22*	sterol sensing domain protein – patch related	Sterol trafficking	2.4	14.7

189282_at	WBGene00007362	*cyp-35C1*	cytochrome P450	Phase 1 enzyme	1.8	54.5

189283_s_at	WBGene00007362	*cyp-35C1*	cytochrome P450	Phase 1 enzyme	2.3	54.7

189512_at	WBGene00015400	*cyp-35A2*	cytochrome P450	Phase 1 enzyme	3.0	33.2

a- WormBase gene identifier

b- Gene names and descriptions were derived from WormBase, release WS189 [46].

c- Gene expression fold change values for dichlorvos SVM predicted high concentration

d- Gene expression fold change values for fenamiphos high concentration

The functions of the other genes differentially affected by the two compounds are poorly described. However, two genes known to be involved with neuronal function are affected differently by dichlorvos and fenamiphos. *ptr-22 *is involved in axon guidance and is more strongly induced by fenamiphos than dichlorvos, and M110.7, the NTE homolog discussed above, appears to respond somewhat differently to the two compounds as well. Finally, F15E6.3 contains an RRM domain which suggests that it may regulate miRNA activity with broader consequences than we have observed in this limited experiment [85].

### Correlation of protein and gene responses

As a whole, the proteins identified as changing in abundance in response to OP exposure respond quite similarly to their transcripts. However, there are five proteins with quantitative changes and four with qualitative changes whose transcript levels do not appear to be changing. There is reason to believe that some of the changes observed only in the proteomics data are substantive. For example, two of the proteins with measured changes (ASP-1 and ASP-5) are cathepsin D homologs [86], and previous work indicates that during starvation cathepsin D undergoes auto-digestion [71].

We found four proteins, which were identified either only in the control or only in the OP samples and which showed no differences in their expression in the microarray analysis. This difference in behavior could result from post-transcriptional regulation, but it might also result from limitations in mass spectrometric analysis. Successful protein identification may not have occurred because the detection of lower abundance peptide ions may have been masked by the presence of higher abundance ones, or because of random effects during the mass spectral analysis. However, the prediction of changes in abundance for five other proteins identified only in OP samples is supported by consistent regulation of the gene transcripts. Thus, while comparisons between conditions must be interpreted cautiously when there is a protein identification in only one of them, mass spectral analysis is a viable means of screening for changes in the abundance of proteins.

## Conclusion

We developed an exposure protocol for comparing the effects of different toxicants with varying mechanisms of action based on the developmental arrest displayed by stressed *C. elegans *worms. Using this technique in conjunction with genomic and proteomic analysis, we identified changes in expression of a group of genes and proteins that are consistent with muscle regeneration or repair resulting from mechanical damage during hypercontraction of muscle in OP exposed worms. In addition we found evidence of cell death stimulated by OP exposure and the induction of (in this limited comparison) OP-specific pathways of detoxification. The effects we observed are similar to those reported in worms exposed to the carbamate, aldicarb, under conventional culture conditions [6,87] and include developmental delays, muscle hypercontraction, reduced mobility, and failure to feed.

Using unsupervised gene ontology analyses of OP specific gene responses, we identified an enrichment of several functional categories of genes plausibly involved in detoxification. We did not observe statistically significant over-representation of any other functional annotation groups, including the ones that we discussed above. Therefore, we cannot rigorously conclude that they occur in our data set more frequently than expected by chance. However, it is possible that more might have been significantly enriched in a larger, less strictly limited set of genes. Further, some actual functional associations may have been missed in the ontology analyses, because available ontologies fail to capture the complexities of some biological functions. Even though we did not identify statistically significant ontology groups for all the functions we considered, the functional categories identified are consistent with the known actions of OPs and provide a foundation for ongoing work elucidating the complete mechanism of OP toxicity.

While we did not pursue changes in gene expression resulting from mefloquine exposure, we found that it was quite easy to discriminate OP-specific responses from ones consequent on mefloquine treatment. Indeed we were able to find differences in gene and protein expression resulting from exposure to the two different OPs in this study, dichlorvos and fenamiphos. These differences seem to indicate that at least somewhat distinct detoxification pathways are induced by the two compounds, likely reflecting their different chemical structures. We also found differences in the expression of two molecules involved in neurological function and of a possible regulator of miRNA activity that differ between the two OPs. These findings suggest that it may be possible to identify "signature" changes in gene expression even for closely related compounds or groups of compounds.

While we originally undertook these experiments partly to identify possible off-target and persistent effects of OP exposure, we did not find clear candidates for this role, perhaps because of the duration of the experiment. Nevertheless, we found alterations in the expression of a carboxylesterase which could affect previously unidentified pathways of intoxication or detoxification and other biological processes. We also found altered expression of a possible regulator of miRNA activity which could ultimately affect the expression of downstream genes. Under the conditions of these experiments, we found only a modest difference in the expression of one of the *C. elegans *NTE homologs; this observation is consistent with observations of human astrocytes exposed to the OP chlorpyrifos, where the NTE gene expression level changes little [88].

The technical approaches we have used in this work have both strengths and weaknesses. Even when proteomic and functional genomic approaches are used together, not all possible biochemical processes and regulatory events that may be important for understanding OP toxicity will be identified. Analyses of the post-translational modifications of proteins, small molecule signaling events, or cell physiological processes would certainly provide an increased understanding of the mechanisms of OP toxicity. However, our approach did reveal OP-specific changes in the expression of a number of molecules of known and unknown function, some or all of which may prove to be critical for our ultimate understanding of the mechanisms of OP toxicity and adaptation. Examining the function of these new players in classically designed studies of mechanism could provide new insights into the overall cell and organismal physiology of OP insult.

## Abbreviations

AChE: acetylcholinesterase; ANOVA: analysis of variance; C: Celsius; EC: effect concentration; EDTA: ethylene diamine tetraacetic acid; EGA: early gravid adult; EGTA: ethylene glycol tetraacetic acid; FDR: false discovery rate; g: gravity; h: hour; HPLC: high pressure liquid chromatography; M: molar; m/z: mass to charge ratio; MAPK: mitogen-activated protein kinase; MAS: Microarray Suite; mg: milligram; min: minute; mL: milliliter; mM: millimolar; mm: millimeter; nAChR: nicotinic acetylcholine receptor; nm: nanometer; NTE: neuropathy target esterase; OP: organophosphorus; PC: principal component; PCA: principal component analysis; PLGS: ProteinLynx Global Server; RMA: robust multi-array averaging; s: second; SVM: support vector machine; UGT: UDP-glucuronosyl/glucosyl transferase; USACEHR: US Army Center for Environmental Health Research; UV: ultraviolet; V: volt; μg: microgram; μL: microliter.

## Authors' contributions

JAL designed experiments, performed experiments, analyzed the data, and wrote the paper. MS designed experiments, performed experiments, and analyzed the data. EG performed experiments. WED performed experiments and wrote the paper. DAJ designed experiments, analyzed data, and wrote the paper. All authors have read and approved the final manuscript.

## Supplementary Material

Additional File 1 **Protein Methods**. This file contains a detailed description of the procedures used for protein processing, mass spectral analysis and proteomic data analysis performed in this work. Click here for file

## References

[B1] Costa LG (2006). Current issues in organophosphate toxicology. Clin Chim Acta.

[B2] Glynn P (2006). A mechanism for organophosphate-induced delayed neuropathy. Toxicol Lett.

[B3] Pope CN, Chakraborti TK, Chapman ML, Farrar JD (1992). Long-term neurochemical and behavioral effects induced by acute chlorpyrifos treatment. Pharmacol Biochem Behav.

[B4] European Centre for Ecotoxicology and Toxicology of Chemicals (1998). Organophosphorus Pesticides and Long-term Effects on the Nervous System Brussels, Belgium.

[B5] Committee on Gulf War and Health (2003). Gulf War and Health: Insecticides and Solvents.

[B6] Nguyen M, Alfonso A, Johnson CD, Rand JB (1995). *Caenorhabditis elegans *mutants resistant to inhibitors of acetylcholinesterase. Genetics.

[B7] Weinbroum AA (2005). Pathophysiological and clinical aspects of combat anticholinesterase poisoning. Brit Med Bull.

[B8] Jokanovic M, Stukalov PV, Kosanovic M (2002). Organophosphate induced delayed polyneuropathy. Curr Drug Targets CNS Neurol Disord.

[B9] Brown MA, Brix KA (1998). Review of health consequences from high-, intermediate- and low-level exposure to organophosphorus nerve agents. J Appl Toxicol.

[B10] Sarin S, Gill KD (1998). Biochemical and behavioral deficits in adult rat following chronic dichlorvos exposure. Pharmacol Biochem Behav.

[B11] Raveh L, Brandeis R, Gilat E, Cohen G, Alkalay D, Rabinovitz I, Sonego H, Weissman BA (2003). Anticholinergic and antiglutamatergic agents protect against soman-induced brain damage and cognitive dysfunction. Toxicol Sci.

[B12] Kamel F, Hoppin JA (2004). Association of pesticide exposure with neurologic dysfunction and disease. Environ Health Perspect.

[B13] Dow GS, Hudson TH, Vahey M, Koenig ML (2003). The acute neurotoxicity of mefloquine may be mediated through a disruption of calcium homeostasis and ER function *in vitro*. Malar J.

[B14] Dow GS, Caridha D, Goldberg M, Wolf L, Koenig ML, Yourick DL, Wang Z (2005). Transcriptional profiling of mefloquine-induced disruption of calcium homeostasis in neurons *in vitro*. Genomics.

[B15] Williams PL, Dusenbery DB (1988). Using the nematode *Caenorhabditis elegans *to predict mammalian acute lethality to metallic salts. Toxicol Ind Health.

[B16] Anton AH, Berk AI, Nicholls CH (1992). The "anesthetic" effect of alcohols and alkanes in *Caenorhabditis elegans *(C.e.). Res Commun Chem Pathol Pharmacol.

[B17] Morgan PG, Sedensky MM (1995). Mutations affecting sensitivity to ethanol in the nematode, *Caenorhabditis elegans*. Alcohol Clin Exp Res.

[B18] Bargmann CI (1998). Neurobiology of the *Caenorhabditis elegans *genome. Science.

[B19] Anderson GL, Boyd WA, Williams PL (2001). Assessment of sublethal endpoints for toxicity testing with the nematode *Caenorhabditis elegans*. Environ Toxicol Chem.

[B20] Cole RD, Anderson GL, Williams PL (2004). The nematode *Caenorhabditis elegans *as a model of organophosphate-induced mammalian neurotoxicity. Toxicol Appl Pharmacol.

[B21] Humphrey JA, Hamming KS, Thacker CM, Scott RL, Sedensky MM, Snutch TP, Morgan PG, Nash HA (2007). A putative cation channel and its novel regulator: cross-species conservation of effects on general anesthesia. Curr Biol.

[B22] Leung MCKL, Williams PL, Benedetto A, Au C, Helmcke KJ, Aschner M, Meyer J (2008). *Caenorhabditis elegans*: An Emerging Model in Biomedical and Environmental Toxicology. Toxicol Sci.

[B23] Reichert K, Menzel R (2005). Expression profiling of five different xenobiotics using a *Caenorhabditis elegans *whole genome microarray. Chemosphere.

[B24] Menzel R, Yeo HL, Rienau S, Li S, Steinberg CE, Sturzenbaum SR (2007). Cytochrome P450s and short-chain dehydrogenases mediate the toxicogenomic response of PCB52 in the nematode *Caenorhabditis elegans*. J Mol Biol.

[B25] Kim Y, Sun H (2007). Functional genomic approach to identify novel genes involved in the regulation of oxidative stress resistance and animal lifespan. Aging cell.

[B26] Pope CN (1999). Organophosphorus pesticides: do they all have the same mechanism of toxicity?. J Toxicol Environ Health B Crit Rev.

[B27] Glynn P (1999). Neuropathy target esterase. Biochem J.

[B28] Lotti M, Moretto A (2005). Organophosphate-induced delayed polyneuropathy. Toxicol Rev.

[B29] Lewis JA, Fleming JT (1995). Basic culture methods. Method Cell Biol.

[B30] Tomlinson GA, Rothstein M (1962). Nematode biochemistry. I. Culture methods. Biochim Biophys Acta.

[B31] Sayre FW, Hansen EL, Yarwood EA (1963). Biochemical aspects of the nutrition of *Caenorhabditis briggsae*. Exp Parasitol.

[B32] Hieb WF, Stokstad EL, Rothstein M (1970). Heme requirement for reproduction of a free-living nematode. Science.

[B33] Vanfleteren JR (1974). Nematode growth factor. Nature.

[B34] Houthoofd K, Braeckman BP, Lenaerts I, Brys K, De Vreese A, Van Eygen S, Vanfleteren JR (2002). Axenic growth up-regulates mass-specific metabolic rate, stress resistance, and extends life span in *Caenorhabditis elegans*. Exp Gerontol.

[B35] Szewczyk NJ, Udranszky IA, Kozak E, Sunga J, Kim SK, Jacobson LA, Conley CA (2006). Delayed development and lifespan extension as features of metabolic lifestyle alteration in *C. elegans *under dietary restriction. J Exp Biol.

[B36] Castelein N, Hoogewijs D, De Vreese A, Braeckman BP, Vanfleteren JR (2008). Dietary restriction by growth in axenic medium induces discrete changes in the transcriptional output of genes involved in energy metabolism in *Caenorhabditis elegans*. Biotechnol J.

[B37] Szilagyi M, Gehman E, Lapenotiere H, Lewis J, Clegg E, Jackson DA (2006). Global Alterations in Gene Expression During Organophosphate Pesticide Intoxication and Recovery: Interim Report. http://handle.dtic.mil/100.2/ADA469210.

[B38] Rao AU, Carta LK, Lesuisse E, Hamza I (2005). Lack of heme synthesis in a free-living eukaryote. Proc Natl Acad Sci U S A.

[B39] Stiernagle T, Hope IA (1999). Maintenance of C. elegans. C elegans: a practical approach.

[B40] Affymetrix – Fluidics Scripts. http://www.affymetrix.com/support/technical/fluidics_scripts.affx.

[B41] Irizarry RA, Bolstad BM, Collin F, Cope LM, Hobbs B, Speed TP (2003). Summaries of Affymetrix GeneChip probe level data. Nucleic Acids Res.

[B42] The R Project for Statistical Computing. http://www.r-project.org/.

[B43] Bioconductor: open source software for bioinformatics. http://www.bioconductor.org/.

[B44] Vapnik VN (1998). Statistical Learning Theory.

[B45] Benjamini Y, Hochberg Y (1995). Controlling the false discovery rate: a practical and powerful approach to multiple testing. J Roy Stat Soc B Met.

[B46] WormBase. http://www.wormbase.org/.

[B47] Dennis G, Sherman BT, Hosack DA, Yang J, Gao W, Lane HC, Lempicki RA (2003). DAVID: Database for Annotation, Visualization, and Integrated Discovery. Genome Biol.

[B48] Huang DW, Sherman BT, Lempicki RA (2009). Systematic and integrative analysis of large gene lists using DAVID bioinformatics resources. Nat Protoc.

[B49] DAVID Bioinformatics Resources 2008. http://david.abcc.ncifcrf.gov.

[B50] Zeeberg BR, Feng W, Wang G, Wang MD, Fojo AT, Sunshine M, Narasimhan S, Kane DW, Reinhold WC, Lababidi S (2003). GoMiner: a resource for biological interpretation of genomic and proteomic data. Genome Biol.

[B51] GoMiner Application Build: 246 Database Build: 2008-04. http://discover.nci.nih.gov/gominer.

[B52] GEO: Gene Expression Omnibus. http://www.ncbi.nlm.nih.gov/geo/.

[B53] Hughes MA, Silva JC, Geromanos SJ, Townsend CA (2006). Quantitative proteomic analysis of drug-induced changes in mycobacteria. J Proteome Res.

[B54] Silva JC, Denny R, Dorschel C, Gorenstein MV, Li GZ, Richardson K, Wall D, Geromanos SJ (2006). Simultaneous qualitative and quantitative analysis of the *Escherichia coli *proteome: a sweet tale. Mol Cell Proteomics.

[B55] National Center for Biotechnology Information. http://www.ncbi.nlm.nih.gov/.

[B56] Hapiak V, Hresko MC, Schriefer LA, Saiyasisongkhram K, Bercher M, Plenefisch J (2003). *mua-6*, a gene required for tissue integrity in *Caenorhabditis elegans*, encodes a cytoplasmic intermediate filament. Dev Biol.

[B57] Li S, Armstrong CM, Bertin N, Ge H, Milstein S, Boxem M, Vidalain PO, Han JD, Chesneau A, Hao T (2004). A Map of the Interactome Network of the Metazoan *C. elegans*. Science.

[B58] Moerman DG, Williams BD, The C. elegans Research Community (2006). Sacromere assembly in *C. elegans *muscle. WormBook.

[B59] Aurelio O, Ghannam K, Kokkinides M, Takii I, Jazayeri S, Kim M, Dang D, Kaskowitz S (2003). Functional characterization of the zig-6 and zig-7 genes [abstract]. 14th International C elegans Conference.

[B60] Tomasetto C, Moog-Lutz C, Régnier CH, Schreiber V, Basset P, Rio MC (1995). Lasp-1 (MLN 50) defines a new LIM protein subfamily characterized by the association of LIM and SH3 domains. FEBS Lett.

[B61] Tjepkema M, Hoppe P, Stout J (2006). The UNC-82 Serine/Threonine Kinase Co-localizes with Intermediate Filaments and Functions in Pharyngeal Muscle Cell Attachment [abstract]. C elegans Development & Evolution Meeting.

[B62] Karabinos A, Schulze E, Schünemann J, Parry DAD, Weber K (2003). *In vivo *and *in vitro *evidence that the four essential intermediate filament (IF) proteins A1, A2, A3 and B1 of the nematode *Caenorhabditis elegans *form an obligate heteropolymeric IF system. J Mol Biol.

[B63] Sieburth D, Ch'ng Q, Dybbs M, Tavazoie M, Kennedy S, Wang D, Dupuy D, Rual J-F, Hill DE, Vidal M (2005). Systematic analysis of genes required for synapse structure and function. Nature.

[B64] Jin JP, Zhang Z, Bautista JA (2008). Isoform diversity, regulation, and functional adaptation of troponin and calponin. Crit Rev Eukaryot Gene Expr.

[B65] Treinin M, Gillo B, Liebman L, Chalfie M (1998). Two functionally dependent acetylcholine subunits are encoded in a single *Caenorhabditis elegans *operon. Proc Natl Acad Sci U S A.

[B66] Catalano A, Romano M, Robuffo I, Strizzi L, Procopio A (2001). Methionine aminopeptidase-2 regulates human mesothelioma cell survival: role of Bcl-2 expression and telomerase activity. Am J Pathol.

[B67] Morales A, Lee H, Goñi FM, Kolesnick R, Fernandez-Checa JC (2007). Sphingolipids and cell death. Apoptosis.

[B68] Halaschek-Wiener J, Khattra JS, McKay S, Pouzyrev A, Stott JM, Yang GS, Holt RA, Jones SJM, Marra MA, Brooks-Wilson AR (2005). Analysis of long-lived *C. elegans daf-2 *mutants using serial analysis of gene expression. Genome Res.

[B69] Syntichaki P, Xu K, Driscoll M, Tavernarakis N (2002). Specific aspartyl and calpain proteases are required for neurodegeneration in *C. elegans*. Nature.

[B70] Luke CJ, Pak SC, Askew YS, Naviglia TL, Askew DJ, Nobar SM, Vetica AC, Long OS, Watkins SC, Stolz DB (2007). An intracellular serpin regulates necrosis by inhibiting the induction and sequelae of lysosomal injury. Cell.

[B71] Hawdon JM, Emmons SW, Jacobson LA (1989). Regulation of proteinase levels in the nematode *Caenorhabditis elegans*. Preferential depression by acute or chronic starvation. Biochem J.

[B72] Petalcorin MI, Joshua GW, Agapow PM, Dolphin CT (2005). The fmo genes of *Caenorhabditis elegans *and *C. briggsae*: characterisation, gene expression and comparative genomic analysis. Gene.

[B73] Newcomb RD, Campbell PM, Ollis DL, Cheah E, Russell RJ, Oakeshott JG (1997). A single amino acid substitution converts a carboxylesterase to an organophosphorus hydrolase and confers insecticide resistance on a blowfly. P Natl Acad Sci USA.

[B74] Casida JE, Quistad GB (2005). Serine hydrolase targets of organophosphorus toxicants. Chem Biol Interact.

[B75] Roninson IB (1987). Molecular mechanism of multidrug resistance in tumor cells. Clin Physiol Bioch.

[B76] Klaassen CD, Lu H (2008). Xenobiotic transporters: ascribing function from gene knockout and mutation studies. Toxicol Sci.

[B77] Berdichevsky A, Guarente L (2006). A stress response pathway involving sirtuins, forkheads and 14–3-3 proteins. Cell Cycle (Georgetown, Tex).

[B78] Baumeister R, Schaffitzel E, Hertweck M (2006). Endocrine signaling in *Caenorhabditis elegans *controls stress response and longevity. J Endocrinol.

[B79] Van Gilst MR, Hadjivassiliou H, Yamamoto KR (2005). A *Caenorhabditis elegans *nutrient response system partially dependent on nuclear receptor NHR-49. Proc Natl Acad Sci U S A.

[B80] Murphy CT, McCarroll SA, Bargmann CI, Fraser A, Kamath RS, Ahringer J, Li H, Kenyon C (2003). Genes that act downstream of DAF-16 to influence the lifespan of *Caenorhabditis elegans*. Nature.

[B81] Garofalo A, Rowlinson MC, Amambua NA, Hughes JM, Kelly SM, Price NC, Cooper A, Watson DG, Kennedy MW, Bradley JE (2003). The FAR protein family of the nematode *Caenorhabditis elegans*. Differential lipid binding properties, structural characteristics, and developmental regulation. J Biol Chem.

[B82] Pinkston-Gosse J, Kenyon C (2007). DAF-16/FOXO targets genes that regulate tumor growth in *Caenorhabditis elegans*. Nat Genet.

[B83] Broeks A, Janssen HW, Calafat J, Plasterk RH (1995). A P-glycoprotein protects *Caenorhabditis elegans *against natural toxins. EMBO J.

[B84] The Agency for Toxic Substances and Disease Registry. http://www.atsdr.cdc.gov/.

[B85] Kedde M, Agami R (2008). Interplay between microRNAs and RNA-binding proteins determines developmental processes. Cell Cycle.

[B86] Tcherepanova I, Bhattacharyya L, Rubin CS, Freedman JH (2000). Aspartic proteases from the nematode *Caenorhabditis elegans*. Structural organization and developmental and cell-specific expression of *asp-1*. J Biol Chem.

[B87] Mahoney TR, Luo S, Nonet ML (2006). Analysis of synaptic transmission in *Caenorhabditis elegans *using an aldicarb-sensitivity assay. Nat Protoc.

[B88] Mense SM, Sengupta A, Lan C, Zhou M, Bentsman G, Volsky DJ, Whyatt RM, Perera FP, Zhang L (2006). The common insecticides cyfluthrin and chlorpyrifos alter the expression of a subset of genes with diverse functions in primary human astrocytes. Toxicol Sci.

