# Supplementary material for: Distinct patterns of gene and protein expression elicited by organophosphorus pesticides in Caenorhabditis elegans
Source: BMC Genomics. 2009 Apr 29;10:202. doi: 10.1186/1471-2164-10-202 (PMC2689271; doi:10.1186/1471-2164-10-202)
Supplement: Additional File 1 — Protein Methods. This file contains a detailed description of the procedures used for protein processing, mass spectral analysis and proteomic data analysis performed in this work. [file 1471-2164-10-202-S1.pdf]

Protein Methods

Purification and processing

Frozen worm droplets from the highest concentrations of fenamiphos and dichlorvos exposures and the unexposed controls were pulverized in liquid N_2 using a pre-chilled mortar and pestle. Worm powder was resuspended in 40 mM Tris base supplemented with 1 mM EGTA and 1 X Protease Inhibitor Cocktail (Cat. # P2714, Sigma-Aldrich, St. Louis, MO). The suspension was sonicated three times with 30 s bursts at 30% power using a Vibra Cell VC50 sonicator (Sonics & Materials Inc., Danbury, CT); samples were held for at least 1 min on ice between cycles. After centrifugation (10,787 x g for 30 min at 4° C), the supernatant, containing the soluble proteins, was collected. Protein concentrations were determined using the Lowry method (RC DC Protein Assay Kit, Bio-Rad, Hercules, CA) with IgG as a standard and UV absorbance at 254 and 280 nm. Four milligrams of each sample was lyophilized and resuspended in 200 µL of 8 M urea, 50 mM ammonium bicarbonate (NH_4HCO_3), and 5 mM dithiothreitol. After a 1-hour incubation at room temperature, 20 µL of 0.5 M iodoacetamide, 50 mM NH_4HCO_3 was added, and samples were incubated at room temperature in the dark for 1 h. The samples were diluted to a final concentration of 1 M urea by adding 50 mM NH_4HCO_3 prior to adding 40 µg of sequencing grade trypsin (Promega, Madison, WI); samples were digested overnight at 37° C.

The digested peptides were desalted using a Bond Elut Jr C-18 column (Varian, Inc., Palo Alto, CA). The column was activated with 10 mL 0.1% TFA (trifluoroacetic acid) in acetonitrile (ACN) and equilibrated with 10 mL of 0.1% TFA. Samples were injected on the column after the addition of 8 µL TFA (0.5% final). The column was washed with 10 mL of 0.1 % TFA. Proteins were eluted sequentially with 0.5 mL 50 % ACN, 0.1% TFA and 0.75 mL 75% ACN, 0.1% TFA. The pooled eluate was dried under vacuum in an Eppendorf Concentrator 5301 (Westbury, NY).

HPLC separation of peptides

Peptides were fractionated using mixed mode ion chromatography. Dried tryptic peptides (see above) were reconstituted in 135 µL of 10% ACN, 20 mM ammonium acetate (NH_4OAc) by vortexing for 1 hour. Samples were clarified by centrifugation prior to injection of 100 µL onto an Agilent 1100 series HPLC system. The reconstituted peptides were separated using a Polycat A 200 x 4.6 mm, 5 µm, 300 Å column, with a 10 x 4 mm guard column and Polywax LP 100 x.4.6 mm, 5 µm, 1000 Å column in series (PolyLC Inc., Columbia, MD); the column was maintained at 35º C. The gradient profile was 20 mM NH_4OAc, 10% ACN to 1.8 M NH_4OAc, 10% ACN in 9 min. The stop time was 17 min and the post time was 20 min using the initial conditions. Time based fractions were collected starting at 2.1 min. The first fraction was collected for 1 min. The next four fractions were collected for 0.5 min, and the last three fractions were collected for 1 min. The diode array detector was set to monitor 280 nm to insure the reproducibility of the separation. Following fractionation, samples were evaporated using an Eppendorf Concentrator 5301 and reconstituted in 60 µL of 3% ACN for analysis by mass spectrometry.

Mass spectrometry

Peptides were analyzed using a nanoACQUITY UPLC coupled to a QTOF Premier quadrupole, orthogonal acceleration time-of-flight tandem mass spectrometer (Waters, Milford, MA). 2 µL of peptides were trapped using a Waters Symmetry C18 180 µm x 20 mm, 5 µm particle size column for 4 min using 0.1% formic acid at 5 µL/min. A Waters BEH C18 75 µm x 100 mm, 1.7 µm particle size analytical column was used at 35° C. Throughout the run, 0.1% formic acid was maintained while varying the ACN concentration as follows: initially 3%, 5% at 1 min, 30% at 101 min, 40% at 105 min, 80% at 111 min, and 3% at 112 min; the analysis was stopped at 122 min. The flow rate was 300 nL/min. Peptides were ionized using electrospray ionization in positive ion mode. Data was collected over the 50-1990 mass to charge (m/z) range with 0.8 s/scan using the Waters

Protein Expression MS^E method, which alternates between low energy scans to survey the precursor ions and high collision energy scans to fragment all of the precursor ions. Computational methods are used to assign fragment ions to precursor ions based on elution profiles [1,2]. Low energy scans were performed with the collision cell at 10 V. The high energy scans were performed while the collision cell ramped from 20 to 40 V. [Glu1]-fibrinopeptide B was used as an external lock mass for accurate mass calculations (m/z = 785.8426); a 1 s lock mass scan was collected every 30 s.

Proteomic data processing

Mass spectrometry data was processed using Protein Lynx Global Server (PLGS) version 2.3 (build 23) with Expression version 2 (Waters). Data preparation parameters were set to manufacturer's default with the exception of a 785.8426 lock mass for charge 2. Workflow parameters for database searches were set to manufacturer's default with the exceptions of a 10% false positive rate, allowing deamidated asparagine and glutamine and oxidated methionine as variable modifications, and enabling PPM calc—which performs a second search using a readjusted mass calibration based on the calculated masses of multiple high scoring identified peptides. An in-house protein identification database was created from all *C. elegans* RefSeq sequences (download date August 8, 2007) [3] combined with likely contaminant proteins including human keratins, bovine albumin, and porcine trypsin. We reduced the overall false positive rate for protein identification to less than 1% by accepting identifications only for proteins identified in at least 2 of 6 replicates for any fraction of any condition. It can be difficult to unambiguously identify closely related proteins, such as splicing isoforms or very similar paralogs which share identical peptides. When definitive identification of an isoform was not possible, all the possible proteins were placed into a "homology group" containing a set of related proteins based on homologs identified in the ion accounting output from PLGS. The ion accounting results from all 96 mass spectrometer runs

were merged to produce a list of all proteins that shared homology with each of the identified proteins. A total of 664 protein homology groups was identified.

Protein quantification

For our investigation of proteins that change in abundance on OP exposure, we combined the high concentrations data sets for dichlorvos and fenamiphos into one group and compared it to the combined unexposed controls for these exposures. We considered only proteins that were identified in at least four replicates of the condition where the protein was at the higher abundance. In addition, we required that the difference in the abundance of the protein between the two conditions be at least 1.5 fold. To obtain final fold differences for each protein of interest we averaged the signals across all replicates and across all fractions in which the protein was identified. Only proteins with an average change greater than 1.5 fold are reported.

For some identified proteins, we were unable to determine quantitative differences across samples because the protein was detected in only one condition. Since mass spectrometric methods used for protein identification suffer from a strong bias towards more abundant proteins, the identification of proteins in one sample but not the other may indicate a difference in abundance. However, failure to identify a protein in a sample does not necessarily indicate an absence of that protein. The protein could be masked by other proteins of higher abundance, or random effects within the run might have affected the quality of the peptide identifications. We have chosen to report proteins which were identified in at least four replicates of a single fraction in one condition but absent in the other as experiencing changes in abundance caused by the OP exposure because MS^E is less prone to sampling effects than tandem mass spectrometric methods and because the overall amounts of proteins across samples were consistent.

References

1. Hughes MA, Silva JC, Geromanos SJ, Townsend CA: **Quantitative proteomic analysis of drug-induced changes in mycobacteria.** *J Proteome Res* 2006, **5:**54-63.

2. Silva JC, Denny R, Dorschel C, Gorenstein MV, Li GZ, Richardson K, Wall D, Geromanos SJ: **Simultaneous qualitative and quantitative analysis of the *Escherichia coli* proteome: a sweet tale.** *Mol Cell Proteomics* 2006, **5:**589-607.

3. **National Center for Biotechnology Information** [http://www.ncbi.nlm.nih.gov/]
